# Infectious Disease as a Modifiable Risk Factor for Dementia: A Narrative Review

**DOI:** 10.3390/pathogens13110974

**Published:** 2024-11-07

**Authors:** Thomas J. Farrer, Jonathan D. Moore, Morgan Chase, Shawn D. Gale, Dawson W. Hedges

**Affiliations:** 1 Idaho WWAMI Medical Education Program, University of Idaho, Moscow, ID 83844, USA; jonathandm@uidaho.edu; 2 The Neuroscience Center, Brigham Young University, Provo, UT 84602, USA; mchase11@student.byu.edu (M.C.); shawn_gale@byu.edu (S.D.G.); dawson_hedges@byu.edu (D.W.H.); 3 The Department of Psychology, Brigham Young University, Provo, UT 84602, USA

**Keywords:** infectious disease, dementia prevention, neuroinflammation, vaccinations, antiviral treatment

## Abstract

This narrative review examines infectious diseases as modifiable risk factors for dementia, particularly in the context of an aging global population. As the prevalence of Alzheimer’s disease and related dementias is expected to rise, prevention has become increasingly important due to the limited efficacy of current treatments. Emerging evidence links specific infectious diseases to increased dementia risk, possibly through mechanisms like neuroinflammation and disruption to normal cell function. Here, we review findings on how viral and bacterial infections contribute to dementia and explore potentially preventive measures, including vaccinations and antiviral treatments. Studies indicate that vaccinations against influenza, herpes zoster, and hepatitis, as well as antiviral treatments targeting human herpesvirus, could reduce the incidence of dementia. Additionally, non-pharmaceutical interventions during pandemics and in long-term care settings are highlighted as effective strategies for reducing the spread of infectious diseases, potentially lowering dementia risk. Putative mechanisms underlying the protective effects of these interventions suggest that reducing systemic inflammation may be important to their efficacy. While the currently available evidence suggests at best an association between some infectious diseases and dementia, this narrative review emphasizes the need to incorporate infectious disease prevention into broader public health strategies to potentially mitigate the growing burden of dementia. Further research is required to explore these preventive measures across diverse populations and to deepen our understanding of the biological mechanisms involved.

## 1. Introduction

The average age of the global population is steadily increasing [1]. As an example, in the United States, all baby boomers will be over 65 years old by 2030. It is estimated that by 2034, older adults will outnumber children for the first time in the United States, and one in five Americans will be over 65 years old [2]. The aging population is at increased risk of chronic health conditions and normal aging factors, such as adverse musculoskeletal, sensory–perceptual, and immunologic changes. The incidence of dementia also increases with age. Characterized by declines in cognition that negatively impact function and independence in daily living, Alzheimer’s disease (AD) and related dementias are a significant public health concern and contribute to mortality and morbidity. The Alzheimer’s Disease International organization estimates that 55 million people worldwide currently live with dementia, but this number is projected to reach 78 million by 2030 and 131 million by 2050 [3,4]. One potential contributing factor to dementia is infectious disease, with vaccines potentially being protective for dementia. Infections remain a public health concern in the older population and are associated with longer hospital stays, higher medical costs, and higher all-cause and attributable mortality [5,6,7,8]. Additionally, hospital-acquired infections are more common in elderly patients, with 7.4% of individuals under age 65 experiencing healthcare-associated infections, but 11.5% of those older than age 85 experiencing healthcare-associated infections [9,10]. In approximately 33% of deaths of adults ages 65 years and older, hospital-acquired infections are the primary cause of death [9]. In addition to increased mortality and morbidity, viral and non-viral infectious diseases have also been associated with dementia. In this narrative review, we first review the association between infectious diseases and dementia and then examine how vaccinations, antiviral treatments, and non-pharmacologic interventions may reduce dementia incidence.

## 2. A Focus on Prevention

While clinical trials for dementia treatment are ongoing [11], the available medications for AD, including the monoclonal antibodies aducanumab, lecanemab, and donanemab, have shown limited efficacy [12]. Because of this, a growing body of research literature has focused on the prevention of dementia [13,14]. A variety of risk factors, including ones that are potentially modifiable, have been associated with dementia, including AD. Among the potentially modifiable risk factors associated with dementia are depression, diabetes, exposure to air pollution, a history of head injury, hearing loss, hypertension, increased cholesterol, obesity, vision loss, elevated alcohol use, decreased social interaction, low educational attainment, physical inactivity, and smoking. These risk factors collectively may account for close to half of all cases of dementia [13]. Accordingly, modification of these risk factors has the potential to substantially reduce incident dementia [13]. Certain infectious diseases, including herpes simplex virus types 1 and 2 (HSV-1; HSV-2) [15], have also been associated with dementia and could be other potentially modifiable risk factors for dementia. The importance of infectious diseases in dementia risk compared to other factors is difficult to determine as most research focuses on the increased risk of infectious diseases in dementia and not on the proportion of dementia that is caused by infectious diseases; however, Livingston et al.’s (2024) 14 factors were estimated to be responsible for 50% of dementia cases, leaving the remaining 50% due to other factors [13]. Research demonstrating that infectious disease is a strong predictor of dementia supports the possibility that at least some portion of the remaining risk could be due to infectious diseases.

### 2.1. Viral Pathogens That Have Been Associated with Dementia

A number of viruses have been associated with incident dementia (Table 1). Human immunodeficiency virus (HIV) is estimated to infect approximately 39.9 million people across the globe as of 2023, with 630,000 people dying in 2023 from HIV-related causes [16]. This virus, which affects the lives of so many people, is also thought to be associated with incident dementia. Prior to the advent of combination antiretroviral therapy (cART), HIV was associated with a high rate of dementia, with approximately 50% of individuals with HIV having dementia symptoms or being neurocognitively impaired [17]. Even with cART, HIV may result in mild to moderate declines in cognitive function [18]. Further, epidemiological studies have also identified an increased risk of dementia with HIV. For example, in older veterans, 5% of those living with HIV developed dementia as compared with 3% of those without HIV [18]. A study on a U.S. sample of patients from Kaiser Permanente found a significant association between HIV positivity and dementia (hazard ratio (HR): 1.58, 95% CI: 1.31–1.92) [19]. In contrast, a cross-sectional study conducted in South Africa among an older sample reported an 18.18% (10/55) dementia prevalence in HIV-positive participants compared to 10.68% (117/1095) prevalence in HIV-negative participants (*p*: 0.131) [20]. With the increasing population of individuals older than 50 with HIV [20], it will be important to better understand the relationships between HIV, aging, and antiretroviral therapy so that affected individuals, including those in low and middle-income countries, can receive the best possible treatment with the hopes of improving their quality of life.

#### Synergistic Effect of Multiple Viruses in Association with Dementia

According to the World Health Organization 2024 Global Hepatitis Report, 50 million people have hepatitis C virus (HCV) infection, another viral disease that has been associated with incident and prevalent dementia [21,23,28]. As of 2020, HSV-1 had an estimated global prevalence of 64.2% for those under the age of 50 years [29]. The association between HSV-1 and dementia is currently a developing area of research, with findings showing associations between HSV-1 and dementia [30,31], although not all findings have been consistent. For example, while Murphy et al. (2021) [22] found a significant association between HSV-1 positivity and a decline in memory, executive function, information processing, and global cognition, they did not find an association between HSV-1 seropositivity and Alzheimer’s disease. In addition to HSV-1, Beydoun et al. (2024) [23] found in a retrospective longitudinal design of up to 30 years that baseline HSV-2 seropositivity increased dementia risk.

Associated with multiple neurocognitive and neuropsychiatric conditions [32], cytomegalovirus (CMV) has an estimated global prevalence of 83% of the general population [33] and has been associated with AD [24], vascular dementia, and general dementia [25]. Other viruses that have been associated with dementia include human papillomavirus [26], with an estimated global prevalence of 31% in males [34] and prevalence estimates ranging from 14 to 24% for women depending on the region [35], and varicella-zoster virus (VZV), with an estimated global incidence of 83,963,744 in 2019 [27,36], although not all findings have been consistent [37].

In addition to associations between individual viruses and dementia, there may be synergistic effects of multiple viruses on dementia risk. A South Korean cohort of 752,205 participants evaluated the association between both VZV and HSV with dementia [38]. In this study, both VZV (HR: 1.52, 95% CI: 1.46–1.57) and HSV (HR: 1.49, 95% CI: 1.42–1.56) were associated with a higher risk of AD [38], and both VZV (HR: 1.34, 95% CI: 1.16–1.54) and HSV (HR: 1.46, 95% CI: 1.24–1.72) were associated with a higher risk of vascular dementia [38]. Moreover, co-infection with both VZV and HSV resulted in a higher risk than either infection separately for AD (HR: 1.75, 95% CI: 1.66–1.85) and vascular dementia (HR: 1.52, 95% CI: 1.24–1.87) [38]. Furthermore, a case–control study comparing AD cases and corresponding controls for prior infection of HSV-1 and CMV reported a significant interaction between CMV and HSV-1 in the development of AD (odds ratio (OR): 5.662, *p*: 0.007) [39].

### 2.2. Non-Viral Pathogens

In addition to viral infections, other pathogens have been associated with dementia, including the parasite *Toxoplasma gondii* [40,41,42] and the bacterial diseases neurosyphilis [43] and Lyme disease [44,45] (Table 2). The bacterium *Helicobacter pylori* has been associated with dementia [46,47,48,49]. It is estimated that approximately 4.3 billion people live with *H. pylori* across the globe [50]. Among older adults already diagnosed with AD, eradication therapy for *H. pylori* was associated with slower disease progression relative to those who did not receive eradication therapy (OR: 0.35; 95% CI: 0.23–0.52) [51]. Similarly, a 2-year longitudinal study demonstrated that individuals with AD have higher baseline rates of *H. pylori* compared to controls (88% versus 46.7%) and that eradication therapy improved both cognitive and functional test results in cases with treatment success [52].

As with viral infections, there may be a role for bacterial combinations in dementia rather than a single species [63]. For example, periodontal disease is associated with an increased risk of AD [57]. Periodontal disease is typically a chronic polymicrobial infection and is common among older adults, with one epidemiological study suggesting that up to 64% of adults over the age of 65 have moderate to severe chronic periodontitis [64]. Additionally, among older adults with normal cognitive function, periodontal disease is associated with an elevated level of beta-amyloid in the brain, as measured with positron emission tomography scanning [65]. Antibodies for periodontal pathogens are also present in the cerebrospinal fluid of older adults multiple years before AD onset [66].

In their meta-analysis, Bouziane, Lattaf, and Maan (2023) found a consistent association between periodontal disease and AD [53]. The authors suggested that this is likely due to increased systemic inflammation and pro-inflammatory cytokines. In a large population-based cohort study, Beydoun and colleagues (2020) found that multiple periodontal pathogens increase the risk for AD and mortality [67]. Asher et al. (2022) [54] found that periodontal disease is associated with both cognitive decline (OR: 1.23, 95% CI: 1.05–1.44) and an increased risk of dementia (HR: 1.21, 95% CI: 1.07–1.38) [54]. A meta-analysis by Nadim et al. (2020) [68] likewise found an elevated relative risk of dementia among those with periodontal disease. Specifically, among all studies included in their analysis, the risk of dementia was 38% higher for those with periodontal disease. Further, in a sub-analysis of case-controlled studies, the risk was far higher, with a 125% increased risk of dementia (risk ratio (RR): 2.25, 95% CI: 1.48–3.43) [68]. Most recently, an additional meta-analysis [69] had similar findings, including an increased risk of cognitive impairment and dementia in older adults with periodontal disease.

While the association between periodontal disease and dementia is still being explored, the available evidence suggests that treatment or prevention of these types of oral diseases may mitigate population incidence of dementia. The Nadim et al. study [68] included a population-attributable risk analysis, which estimates the number of cases of dementia that would not occur if steps were taken to reduce periodontal disease. The findings suggested that a 50% reduction in the current prevalence of periodontal disease would result in 850,000 fewer cases of dementia and that a 75% reduction would result in 1.29 million fewer cases of dementia [68].

Related to the associations between periodontal disease and dementia are associations between the composition of the oral microbiome and cognitive function. Specifically, a lower diversity of bacteria in the oral microbiome is associated with cognitive decline [68]. Indeed, specific bacteria in the oral microbiome have been linked with cognitive decline, including *Porphyromonas gingivalis* [55,70], *Tannerella forsythia* [55], *Streptococcus mutans* [56], *Actinobacillus actinomycetemcomitans* [58], *Fusobacterium nucleatum* [59], and *Treponema denticola* [55]. Patients with the highest level of *Porphyromonas gingivalis* in blood, compared to those with the lowest, were more likely to exhibit poorer delayed verbal recall [70]. Additionally, the presence of *Porphyromonas gingivalis* in saliva was associated with poorer performance on the Mini-Mental State Examination (MMSE) [55]. *Treponema denticola* and *Tannerella forsythia* may affect the immune response in patients with AD due to lower concentrations of inflammatory markers [55]. Similarly, the presence of *Streptococcus mutans* in saliva has been associated with a greater incidence of cerebral microbleeds [56], and *Actinobacillus actinomycetemcomitans* produces lipopolysaccharides and leukotoxins associated with increased neuroinflammation [57,58]. In vivo experiments observed that *Fusobacterium nucleatum* can lead to worsened symptoms of AD in mice, including greater accumulation of beta-amyloid, tau protein phosphorylation, and cognitive impairment [59].

In addition to associations between the oral microbiome and cognitive function, there may be associations between the intestinal microbiome and dementia. In patients with dementia, compared with those without dementia, the level of enterotype I bacteria was lower while the level of enterotype III was higher [71]. Intestinal microbiome diversity in people with AD was significantly lower than in people without AD [72].

#### 2.2.1. Viral Encephalitis and Bacterial Meningitis

Viral encephalitis increases the risk of developing dementia; researchers reported that individuals who had viral encephalitis had a 20 times higher likelihood of being diagnosed with AD than individuals who did not have viral encephalitis [73]. Bacterial meningitis causes neuroinflammation leading to damage to neurons with permanent changes to neuronal circuits that can lead to dementia [74].

#### 2.2.2. Pneumonia and Sepsis

Infectious diseases in older age, such as sepsis and pneumonia, are linked with the development of dementia. Sepsis has an estimated incidence of 677.5 per 100,000 globally [75]. Additionally, the leading infection that causes sepsis is pneumonia, specifically community-acquired pneumonia [76]. The estimated incidence for community-acquired pneumonia seems to vary by age of individuals, with older populations having higher incidence rates and country, but many prevalence rates seem to fall between 24.8 and 209.3 cases per 10,000 [76]. A meta-analysis of eight studies reported that having severe sepsis increased odds of cognitive impairment and dementia [61]. In vivo models suggest that sepsis increases biomarkers for neuroinflammation and may result in persistent damage to the brain [62]. Additionally, up to 70% of people who experience sepsis develop delirium, the severity and duration of which are associated with the risk of AD and related dementias [62]. Furthermore, bacterial pneumonia has been found to increase the risk of dementia, including vascular dementia, AD, and unspecified dementia [60]. In particular, patients who had bacterial pneumonia had a 2.44 times higher risk of developing AD, a 4.15 times higher risk of developing vascular dementia, and a 2.62 times higher risk of developing unspecified dementia [60].

## 3. Infectious Disease Prevention to Reduce Dementia Risk

Some viral, bacterial, and parasitic infections can worsen cognitive function and increase the risk of dementia over time [77,78]. While direct evidence specifically linking infectious disease prevention to reduced dementia risk is still emerging, several studies have investigated related factors that can shed light on this topic. Here, we review the literature on associations between vaccinations, antiviral medications, and additional public health interventions and dementia incidence (Table 3).

### 3.1. Vaccinations

Vaccination against certain infectious diseases has been associated with a reduced risk of subsequent dementia and suggests an important means of reducing dementia risk. A recent meta-analysis [79] examined the risk of dementia among recipients of a herpes zoster vaccination. Across the included source studies, the pooled odds ratio was 0.84, suggesting a 16% reduction in dementia risk among those who were vaccinated. Similarly, Wu et al. (2022) [80] conducted a meta-analysis of dementia risk after vaccination of several conditions, and while not all vaccination types demonstrated evidence of reduced dementia risk, the overall effect suggested a 35% reduction in dementia (hazard ratio (HR): 0.65, 95% CI: 0.60–0.71). Specific vaccinations demonstrating reduced dementia risk in this meta-analysis included influenza (HR: 0.74), herpes zoster (HR: 0.69), tetanus, diphtheria, and pertussis (Tdap; HR: 0.69), rabies (HR: 0.43), hepatitis A (HR: 0.78), hepatitis B (HR: 0.82), and typhoid (HR: 0.80) [80]. This study also demonstrated a dose–response relationship: more vaccination types and a greater number of annual influenza vaccinations resulted in significantly reduced dementia risk [80].

While the meta-analysis by Wu and colleagues [80] demonstrated a reduced risk of dementia following vaccination for several viral pathogens, their analysis was equivocal for tuberculosis vaccination (i.e., Bacille Calmette-Guérin (BCG) vaccine). However, several studies have examined whether BCG lowers incident dementia in patients with bladder cancer, as BCG is a standard treatment for such patients. Across five studies, the risk of dementia was consistently lower among those receiving BCG treatment [84,85,86,87,88]. Subsequent meta-analytic examination of these data demonstrated a combined HR of 0.55 (95% CI: 0.42–0.73), suggesting a 45% reduced risk of dementia in bladder cancer patients who received BCG treatment [96]. Additionally, Gofrit et al. [97] found that rates of AD are lower in countries with higher population BCG coverage and suggested that this may be related to protective mechanisms of this vaccine. Specifically, they noted that BCG increases interleukin-2 (IL-2), which promotes the proliferation of regulatory T cells (Treg cells), which have neuroprotective properties [98,99].

Two recent meta-analyses examined dementia risk following influenza vaccination. Veronese et al. (2022) [82] found a 29% reduced risk of dementia an average of 9 years after vaccination of influenza (relative risk ratio (RR): 0.71, 95% CI: 0.60–0.94). Similarly, Sun, Liu, and Liu (2023) [83] reported a 31% reduction in incident dementia following vaccination (RR: 0.69, 95% CI: 0.57–0.83).

In a large population sample in Taiwan, Chen et al. (2018) [92], while identifying a slightly increased risk of dementia among those with herpes zoster (shingles) (HR: 1.11; 95% CI: 1.04–1.17), also demonstrated that individuals prescribed antiviral therapy were at a decreased risk of dementia (HR: 0.55; 95% CI: 0.40–0.77). In a population cohort in Wales (n = 336,341), vaccination for VZV was associated with a reduced risk of dementia (HR: 0.72; 95% CI: 0.69–0.75) [89]. This study also demonstrated a differential impact on dementia type, with a 19% reduced risk of AD but a 34% reduced risk of vascular dementia [89]. Similarly, a population-based cohort study in the UK [81] demonstrated that vaccines for herpes zoster and influenza both reduced the risk of dementia (HR: 0.78; 95% CI: 0.77–0.79 and HR: 0.96; 95% CI: 0.89–0.92, respectively). Of note, these prior studies on shingles vaccination have largely focused on live attenuated varicella-zoster virus, which triggers the immune system to recognize and fight the virus when present. Conversely, a recombinant vaccine contains a protein (glycoprotein E) from the virus, which helps the immune system recognize the virus. A recent population-based study examined the relative difference in dementia risk following either a live or recombinant vaccine for shingles [100] and found that the recombinant vaccine was superior to the live variant in prolonging survival time (time to dementia onset; restricted mean time lost (RMTL) ratio: 0.83, 95% CI: 0.80–0.87) and postulated that the recombinant shingles vaccine serves as an immunostimulant, possibly mitigating risk over time.

While studies investigating the associations between vaccination and risk of subsequent dementia are ongoing, the available findings suggest that vaccines against herpes zoster [79], influenza [80,82], tetanus, diphtheria, and pertussis in the Tdap vaccine, rabies, hepatitis A, hepatitis B, typhoid [80], tuberculosis [96], and VZV [89] may decrease the risk of incident dementia. Moreover, the effect sizes of the reduction of dementia risk are not trivial, with results from meta-analyses showing reductions in dementia risk of 35% [80], 45% [96], and 29% [82]. Together, these findings suggest that vaccination may have considerable preventive potential against dementia. There is additional evidence for reduced dementia following other vaccines, although based on fewer studies. For example, examining two large archival health record databases, Scherrer et al. (2021) [101] found that those who received Tdap vaccination were 42% less likely to have a dementia diagnosis (HR: 0.58). Additional research investigating associations between vaccination and incident dementia is needed, examining these associations in other populations globally. Research into associations between other vaccines and dementia incidence and the mechanisms underlying these associations is also needed.

#### Vaccine Surveillance and Coverage

Despite vaccine safety and mounting evidence of reduced transmission and lowered morbidity following vaccinations [102], many adults remain unvaccinated. Srivastav et al. (2023) [103] reported that around 40% of adults in the United States are hesitant to receive the influenza vaccine, believing there may be some degree of harm from the vaccine or not trusting its efficacy. Recent literature on vaccination coverage for common endemic illnesses suggests that the population vaccination rates are far below national vaccination goals. For example, Lu et al. (2021) [104] reported that only 45.4–46.1% of adults received the influenza vaccine in the 2016 to 2018 seasons. Estimated population coverage was also low for pneumococcal vaccine (69%), herpes zoster (24.1–34.5%), tetanus (62%), Tdap (31.2%), hepatitis A (11.9%), and HPV (52.8%). A 2018 report indicated that influenza coverage was consistently low in Europe, with only 45.5% of older adults receiving the vaccine [105]. Black and colleagues (2023) [106] reported that as of December 2023, only 42.2% of adults had received the seasonal influenza vaccine, and only 18.3% had an updated COVID-19 booster. They also reported that only 17% received a respiratory syncytial virus (RSV) vaccine [106].

One potential cause for these low vaccine coverage rates is vaccine hesitancy. A large barrier to eliminating disease and improving quality of life, vaccine hesitancy also prevents the elimination of some disease-related modifiable risk factors for dementia. The World Health Organization (WHO) defines vaccine hesitancy as being unwilling, either through refusal or delaying acceptance, to receive vaccines, despite those vaccines being available in the community [107]. Some of the primary factors for vaccine hesitancy fall into three broad categories: complacency, confidence, and convenience [107]. Vaccine hesitancy because of complacence often comes from people not seeing the disease the vaccine is protecting against as an issue [107,108]. Confidence issues include lack of trust in the safety of the vaccine and in the people or organizations promoting and creating the vaccine [107,108]. Problems with convenience, such as poor vaccine accessibility, can also result in vaccine hesitancy [107,108]. Two additional factors associated with vaccine hesitancy are communication and context. Providing the necessary information to make informed decisions about receiving vaccines is important, as is understanding the context of individuals receiving the vaccine, including their cultural, ethnic, or religious backgrounds [108]. By taking these many factors of vaccine hesitancy into account, researchers can develop a more complete understanding of the low vaccine coverage rates.

These low coverage rates raise important questions about population risk of dementia. Gofrit et al. (2019) [97] found that rates of AD are lower in countries with higher population BCG vaccine coverage. Similarly, Lehrer and Rheinstein (2022) [109] found higher rates of Parkinson’s disease in US states with lower rates of shingles vaccine coverage. This may support efforts to increase vaccination coverage in an effort to lower dementia incidences [109]. While not specific to dementia prevention per se, several population health studies have explored measures to improve vaccination coverage. For example, vaccine co-administration is an effective strategy for increasing coverage (e.g., a combination of COVID-19 and influenza and a combination of diphtheria, tetanus, and acellular pertussis (Tdap) vaccine [110]). Other effective strategies include vaccine campaigns, reducing the chances for a missed opportunity, point-of-care reminders, and patient education [111,112]. Based on the available literature on vaccination status and lowered dementia risk, we argue that higher vaccination coverage could result in lower rates of incident dementia.

### 3.2. Treatment of Infectious Diseases

Some infectious diseases have behavioral interventions or very accessible treatment options, making those that are risk factors for dementia modifiable risk factors. Periodontal disease is one example of such an infectious disease. Behavioral interventions for periodontitis include smoking cessation, diabetes management, and regular oral-health practices [113]. Medical interventions for periodontal disease are not exhaustively covered here, but it is noted that such intervention in older adults is as effective as it is in younger adults and also reduces the risk of periodontal disease over time [114].

Other infectious diseases can be prevented through vaccination or treated through antiviral treatment. Accumulating research findings have not only seen associations between vaccination and dementia risk but have also identified associations between antiviral treatment and reduced risk of dementia. A recent meta-analysis [90] reaffirmed the association between VZV and increased dementia risk but also demonstrated that those who undergo antiviral treatment have a reduced risk of subsequent dementia (HR: 0.84; 95% CI: 0.71–0.99). In a matched cohort design [91], dementia risk was examined among those diagnosed with the HSV or VZV and those who had or did not have antiviral treatment. The risk of dementia for those with herpes who did not have antiviral treatment was higher (HR: 1.50) than the risk for those with herpes who did undergo treatment (HR: 0.89), with no observed difference based on the specific type of medication (i.e., acyclovir versus valacyclovir) [91].

Additionally, in another recent meta-analysis, Thapa et al. (2024) [93] examined the risk of dementia following herpes zoster and the differential impact between antiviral treatment versus no treatment. Among the 538,531 patients across source studies, the risk of dementia among those who did not have herpes zoster antiviral treatment was increased (HR: 1.15; 95% CI: 1.03–1.28). However, among those who did undergo antiviral treatment, there was a reduced risk of dementia (HR: 0.68; 95% CI: 0.59–0.77) [93].Weinmann et al. (2024) [115] found somewhat contradictory findings in their study examining the risk of dementia from herpes zoster in an archival sample from a health record database. Specifically, these authors suggested the risk of dementia was not associated with herpes zoster overall (HR: 0.99, 5% CI:0.93–1.05). However, their subsequent analysis found that individuals prescribed antiviral medication had a lower risk of dementia (HR: 0.79; 95% CI: 0.70–0.90). This is consistent with findings by Young-Xu and colleagues (2021) [94], who examined HSV1/HV2 and anti-herpetic treatment in an archival sample of US veterans. While their analysis suggested HSV did not increase the risk of dementia (HR: 0.80), they found that among those who had HSV and underwent anti-herpetic treatment, there was a significant decrease in incident dementia (HR: 0.75; 95% CI: 0.72–0.78) [94].

Tzeng et al. (2018) [95] similarly examined a population healthcare database (n = 33,448) for an association between HSV and dementia and the impact of antiviral treatment. Their findings suggested a substantially increased risk of all-cause dementia among those with HSV, with a hazard ratio of 2.56 (95% CI: 2.35–2.79). Furthermore, among a subsample treated with anti-herpetic medications, there was a reduced risk of dementia over a 10-year follow-up period (HR: 0.092; 95% CI: 0.079–0.108) [95]. Some research suggests that higher cumulative antibiotic use over time may be associated with the risk of dementia [116]. While the mechanism is unclear, it has been suggested it may be due to a negative impact on the gut microbiota [117] and that this effect could depend on the type of antibiotic [118]. However, one study found that sporadic use of antibiotics was associated with a decreased risk of dementia [119]. Relatedly, one small study found that antibiotic treatment for leptospirosis was associated with a decreased risk of dementia and no treatment was associated with an increased risk [120]. Thus, targeted antibiotic treatment of specific pathogens may be protective against dementia.

Together, findings from studies investigating the effects of antiviral medication for viruses suspected of being associated with dementia, such as VZV and HSV, indicate that antiviral treatment could lower the risk of dementia, suggesting that antiviral treatment may have a preventive effect against dementia. In this regard, it is important to note that clinical trials of valacyclovir (an antiviral treatment for herpes viruses) are underway for early AD as a treatment for dementia prevention [121]. In summary, there is emerging evidence that vaccinations and antiviral treatments confer protective effects from dementia and increase survival time. The mechanisms of action appear to be complex and may be related to reduced viral load and viral replication, thus reducing systemic inflammation and penetrance to the CNS.

### 3.3. Potential Mechanisms of Action Underlying Associations Between Vaccination and Antiviral Treatment and Reduced Risk of Incident Dementia

A variety of mechanisms could underlie the associations between infectious diseases and dementia and provide insight into how vaccination and antiviral and antibiotic treatment might reduce the risk of incident dementia. One proposed mechanism for associations between infection and increased risk of dementia suggests that the central nervous system is permeable to some degree and that pathogens may enter the brain and have direct deleterious effects on neural tissue, resulting in neuroinflammation, apoptosis, and dysregulation of cell function and extracellular vesicle function [122]. An indirect pathway occurs when an infection spreads from a peripheral infection to general circulation and subsequently reaches the central nervous system. Regardless of the pathway, the immune response triggers the activation of cytokines, reactive oxygen species, and other agents that disrupt or damage cell function [122].

Several studies have explored potential mechanisms of action for how vaccines and antiviral medications reduce dementia risk, focusing on the same causal mechanism of how viral infection could increase dementia risk in the first place. For instance, studies show that vaccines and antiviral treatment lower viral load, which can reduce levels of systemic inflammation. Specifically, antiviral treatment with cidofovir reduced the viral load of HPV [123], and vaccination for COVID-19 reduced viral load and increased viral clearance [124,125]. Antiviral treatment also mitigates herpes viral reactivation, which would also reduce the frequency of inflammatory reactions [91]. Among those with HIV, combination antiretroviral therapy (cART) reduced viral replication as well as neuroinflammatory processes, and cART reduced rates of dementia in this patient group [126]. Vaccination against or timely antiviral treatment of HSV can prevent the virus from entering or reactivating in the CNS, thereby reducing the direct viral damage to neurons [127]. As noted, et al. (2019) [88] suggested that the lower rates of AD among those receiving BCG vaccination may be related to protective mechanisms of this vaccine. Specifically, they note that BCG increases interleukin-2, which promotes the proliferation of regulatory T cells (Treg cells), which have neuroprotective properties [98,99].

Additionally, there are multiple lines of evidence that vaccinations and antiviral treatments promote cross-reactivity to other pathogens. For example, exposure to one pathogen or its corresponding vaccine can result in heterologous immunity, whereby the immune response generated by the vaccine confers protection against other pathogens that may have similar genetics or similar structures [128]. There is also evidence of epigenetic changes to innate immune cells following vaccinations, including BCG, yellow fever, vaccinia, measles, and influenza. These epigenetic changes result in a trained immunity and allow the body to fight a wider array of pathogens [129]. As an example, the BCG vaccine for tuberculosis provides non-specific protection from other pathogens in both human and animal studies, including yellow fever, human papillomavirus, HSV 1 and HSV 2, respiratory syncytial virus, influenza, hepatitis B, ectromelia virus, vaccinia virus, Japanese encephalitis, and encephalomyocarditis virus [130,131].

### 3.4. Other Behavioral or Public Health Interventions with Potential to Reduce the Incidence of Dementia

While vaccines and antiviral treatments remain a common intervention in disease management, nonpharmaceutical interventions (NPIs) are also effective in reducing the population spread of transmissible infectious diseases, potentially including those that have been associated with dementia. Some examples of NPIs include hand washing, wearing a facemask, quarantining, and social distancing. Studies have examined the effectiveness of such NPIs in reducing death rates in pandemics. Archival studies of NPIs in the 1918–1919 influenza pandemic showed that they did reduce death rates, where interventions included quarantine, staggered business hours, school and church closures, and bans on public gatherings. These studies also examined the impact of how quickly such interventions were implemented [132,133]. Overall, the use of such NPIs reduced death rates across several US cities. Additionally, interventions that were implemented quickly and early resulted in lower peak rates of infection and lower mortality rates [132,133]. The global COVID-19 pandemic provided similar valuable insights into NPI strategies and effectiveness. For example, Pan et al. (2020) [134] examined longitudinal implementation of NPIs early in the COVID-19 pandemic, prior to vaccination availability, and demonstrated reduced rates of transmission based on such interventions as lockdowns, quarantine, and social distancing. Rapid testing allows for a fast assessment of illness status, informing an individual’s decision to quarantine, and thus reducing population spread [135]. School closures, stay-at-home/work-from-home arrangements, and school-closure mandates also reduced the spread of disease [135]. An umbrella review of NPI strategies for reducing respiratory viruses also suggests that physical distancing, masking, and hand hygiene are effective ways to reduce transmission [136]. Importantly, even with pharmaceutical interventions (e.g., influenza or COVID-19 vaccinations), population coverage of these interventions is almost certainly less than 100%, and the continued use of NPIs in conjunction with vaccinations has a synergistic impact on further lowering transmission rates [137].

While public health interventions, such as NPIs, may reduce the spread of disease for certain pathogens, this approach will not necessarily be effective for all contagious diseases. This is because the natural history of any given pathogen will greatly impact its spread. For instance, viruses vary greatly in their incubation periods, the maximum duration of infectiousness, and time to peak infectiousness. As such, a particular virus could spread through a community before symptoms manifest, making strategies for controlling the spread of infection challenging [138]. NPIs can also be effective in reducing transmission of and exposure to infectious diseases outside of pandemics. Among older adults in long-term care facilities, certain NPIs are shown to reduce transmission rates of COVID-19, influenza, and upper-respiratory tract infection [139].

### 3.5. Comparison of Vaccination and Interventions in Dementia Risk Reduction

A meta-analysis including 17 studies found that receiving vaccinations for infectious diseases was associated with a lower risk of dementia (rabies: HR = 0.43; hepatitis B: HR = 0.82; herpes zoster: HR = 0.69; tetanus, diphtheria, and pertussis: HR = 0.69; hepatitis A: HR = 0.78; influenza: HR = 0.74; and typhoid: HR = 0.80) [80]. Additionally, people with more types of vaccinations and those with more yearly influenza vaccinations were less likely to develop dementia [80]. Pooled estimates from this meta-analysis showed that vaccination resulted in a 35% lower risk of dementia (HR = 0.65) [80]. In comparison, other factors associated with a lower risk of dementia, including smoking cessation, cognitive stimulation, and alcohol consumption, tend to have a lower or similar impact on dementia risk. For cessation of tobacco, participants who quit smoking had a lower risk of all-cause dementia (adjusted HR, 0.92; 95% CI: 0.87–0.97) compared with those who maintained their smoking within 20% of their baseline level [140]. In addition, in adjusted analyses, more frequent engagement in adult literacy activities (e.g., letter writing) was associated with an 11% lower risk of dementia (adjusted HR: 0.89; 95% CI: 0.85–0.93). Similarly, engaging in active mental activities (e.g., playing cards) was associated with a 9% lower risk of dementia adjusted (HR: 0.91; 95% CI: 0.87–0.95) [141]. Regarding alcohol consumption, compared to abstinence from alcohol, those who engaged in mild or moderate alcohol consumption had a lower risk of all-cause dementia (adjusted HR [mild]: 0.79; 95% CI: 0.77–0.81; adjusted HR [moderate]: 0.83; 95% CI: 0.79–0.88) [142].

## 4. Conclusions

Accumulating findings suggest associations between infectious diseases and incident dementia [15,143], as well as with other types of neurodegenerative diseases [73]. While the currently available evidence suggests at best an association between some infectious diseases and dementia, these associations provide potentially novel approaches to dementia prevention, particularly given currently low population vaccine coverage globally [104]. In addition to nonpharmaceutical interventions, including during epidemics and pandemics, and public health measures designed to reduce the exposure to and transmission of infectious diseases [136,137], vaccination, antiviral, and antibiotic treatment may reduce incident dementia. Possibly via reducing viral load and inflammation [122], decreasing direct damage to brain tissue [127], promoting heterologous immunity [128], and other as yet unidentified potential mechanisms, vaccination and antiviral treatment may reduce the incidence of dementia. Emerging findings suggest that some of the reductions in dementia risk could be substantial, with reductions in dementia risk according to some studies we review here ranging from 4% [81] to 45% [92,96] depending on the study and virus type. Similarly, the reductions in dementia risk from antiviral treatment could be considerable, with decreased risk in the studies we review here ranging from 16% [90] to approximately 99% [91], again depending on the study and type of virus.

Considerable research needs to be conducted to further evaluate both associations between infectious diseases and dementia and the effects of vaccination, treatment of the diseases themselves, and nonpharmaceutical interventions on dementia incidence. We identify four topics of additional research that could be particularly relevant to understanding the association between infectious diseases and cognitive health in the aging population: (1) understanding the underlying mechanisms and associations between infectious diseases and dementia, including the pathogens and host factors involved; (2) investigating the mechanisms for vaccination and antiviral treatment in reducing incident dementia; (3) researching the relationships between nonpharmaceutical interventions and incident dementia; and (4) using longitudinal studies and clinical trials to investigate how vaccinations and antiviral and antibiotic treatment might affect the cognitive health of aging populations. The accumulating findings showing associations between infectious diseases and incident dementia and early findings suggesting that vaccination and antiviral treatment may reduce incident dementia make infectious diseases important potentially modifiable risk factors for dementia.

## Figures and Tables

**Table 1:** Viral pathogens associated with dementia.

Viral Pathogen	Findings
HIV	Prior to cART, approximately 50% of individuals with HIV developed dementia symptoms or other neurocognitive impairment [17]. In older veterans, 5% of those living with HIV developed dementia as compared with 3% of those without HIV [18]. 58% higher risk of dementia in those HIV positive [19]. In an older South African sample, there was an 18.18% (10/55) dementia prevalence in HIV-positive participants compared to 10.68% (117/1095) prevalence in HIV-negative participants [20].
Hepatitis C	Hepatitis C virus (HCV) infection is associated with incident and prevalent dementia [21].
HSV-1, HSV-2	Murphy et al. (2021) [22] found a significant association between HSV-1 positivity and a decline in memory, executive function, information processing, and global cognition but not with Alzheimer’s disease. Beydoun et al. (2024) [23] found in an up to 30-year retrospective longitudinal design that baseline HSV-2 seropositivity increased dementia risk.
Cytomegalovirus (CMV)	Cytomegalovirus (CMV) has been associated with AD [24], vascular dementia, and dementia [25].
Human papillomavirus	Genital warts caused by human papillomavirus infection are associated with an increased risk of dementia [26].
Varicella-zoster virus (VZV)	Varicella-zoster patients had a higher risk of dementia than those without [27].

**Table 2:** Other pathogens associated with dementia.

Pathogen	Findings
Periodontal Diseases	In their meta-analysis, Bouziane, Lattaf, and Maan (2023) found a consistent association between periodontal disease and AD [53]. Periodontal disease is associated with 1.23 times higher odds of cognitive decline and a 21% higher risk of dementia [54]. Further, in a sub-analysis of case-controlled studies, a greater than double higher risk of dementia was observed for those with periodontal disease.
*Porphyromonas gingivalis*	The presence of *Porphyromonas gingivalis* in saliva was associated with poorer performance on the Mini-Mental State Examination (MMSE) [55].
*Tannerella forsythia*	*Tannerella forsythia* may affect immune response in patients with AD due to lower concentrations of inflammatory markers [55].
*Streptococcus mutans*	*Streptococcus mutans* found in saliva is associated with a higher incidence of cerebral microbleeds [56].
*Actinobacillus actinomycetemcomitans*	*Actinobacillus actinomycetemcomitans* produces lipopolysaccharides and leukotoxins associated with increased neuroinflammation [57,58].
*Fusobacterium nucleatum*	In mouse models, *Fusobacterium nucleatum* has been associated with deteriorating symptoms of AD and with increasing accumulation of beta-amyloid, tau protein phosphorylation, and worsening cognitive impairment [59].
*Treponema denticola*	*Treponema denticola* may affect immune response in AD patients by affecting concentrations of inflammatory markers [55].
Neurosyphilis	Neurosyphilis is associated with progressive dementia [43].
Lyme Disease	Lyme disease has been associated with cognitive impairment [44]. Rare cases have been reported of Lyme disease causing dementia [44,45].
Pneumonia	Bacterial pneumonia has been found to increase the risk of vascular dementia, AD, and unspecified dementia [60]. Patients with bacterial pneumonia had a 2.44 times higher risk of developing AD, a 4.15 times higher risk of developing vascular dementia, and a 2.62 times higher risk of developing unspecified dementia [60].
Sepsis	A meta-analysis reported that severe sepsis increased odds of cognitive impairment and dementia [61]. In vivo research suggests that sepsis increases the presence of biomarkers for neuroinflammation and may result in persistent damage to the brain [62].

**Table 3:** Associations between vaccination and antiviral treatment and dementia.

Vaccine/Antiviral	Findings
Herpes zoster vaccination	In a meta-analysis, those who had been given a herpes zoster vaccination had a lower risk of dementia than those who did not receive the vaccination [79]. Those vaccinated with a herpes zoster vaccination had a lower risk of dementia than those who did not receive the vaccination [80]. A vaccine for herpes zoster reduced the risk of dementia [81].
Influenza vaccination	The more vaccination types and the greater number of yearly influenza vaccinations were associated with reduced dementia risk [80]. A 29% reduced risk of dementia was observed an average of 9 years after vaccination of influenza [82]. A 31% risk reduction in incident dementia was reported after influenza vaccination [83]. A vaccine for influenza reduced the risk of dementia [81].
Tetanus, diphtheria, and pertussis (Tdap) vaccination	Tdap vaccination reduced the risk of dementia [80].
Rabies vaccination	Rabies vaccination reduced the risk of dementia [80].
Hepatitis A vaccination	Hepatitis A vaccination reduced the risk of dementia [80].
Hepatitis B vaccination	Hepatitis B vaccination reduced the risk of dementia [80].
Typhoid vaccination	Typhoid vaccination reduced the risk of dementia [84].
Tuberculosis (Bacille Calmettte-Guérin) vaccination	The risk of dementia was consistently lower for those who had been given the BCG vaccine [84,85,86,87,88].
Varicella-Zoster Virus (VZV) vaccination	Vaccination for VZV was associated with a reduced risk of dementia, with a 19% reduced risk of AD and a 34% reduced risk of vascular dementia [89]. Findings showed VZV increased dementia risk but antiviral treatment reduced subsequent risk of dementia [90].
Herpes antiviral treatment	Those with herpes who did not receive antiviral treatment had a higher dementia risk than those with herpes who did undergo treatment [91]. A slightly increased risk of dementia among those with herpes zoster (shingles) was observed, but after treatment with antiviral therapy, there was a decreased risk of dementia [92]. The risk of dementia among those who did not have herpes zoster antiviral treatment was increased. However, those who underwent antiviral treatment had a reduced risk of dementia [93]. Those with HSV who underwent anti-herpetic treatment had reduced incident dementia [94]. An increased risk of all-cause dementia was observed among those with HSV. However, those treated with anti-herpetic medications had a lower risk of dementia [95].
